# Machine Learning–Based Prediction of Clinical Outcomes in Patients With Cancer Receiving Systemic Treatment Using Step Count Data Measured With Smartphones

**DOI:** 10.1200/CCI-25-00023

**Published:** 2025-06-30

**Authors:** Calvin G. Brouwer, Branca M. Bartelet, Joeri A.J. Douma, Leni van Doorn, Evelien J.M. Kuip, Henk M.W. Verheul, Laurien M. Buffart

**Affiliations:** ^1^ Department of Medical BioSciences, Radboud University Medical Center, Nijmegen, the Netherlands; ^2^ Department of Internal Medicine, Medical Centre Leeuwarden, Leeuwarden, the Netherlands; ^3^ Department of Medical Oncology, Erasmus MC Cancer Institute, University Medical Center Rotterdam, Rotterdam, the Netherlands; ^4^ Department of Medical Oncology and Department of Anesthesiology, Pain and Palliative Care, Radboud University Medical Center, Nijmegen, the Netherlands

## Abstract

**PURPOSE:**

This study aimed to investigate whether changes in step count, measured using patients' own smartphones, could predict a clinical adverse event in the upcoming week in patients undergoing systemic anticancer treatments using machine learning models.

**METHODS:**

This prospective observational cohort study included patients with various cancer types receiving systemic anticancer treatment. Physical activity was monitored continuously using patients' own smartphones, measuring daily step count for 90 days during treatment. Clinical adverse events (ie, unplanned hospitalizations and treatment modifications) were extracted from medical records. Models predicting adverse events in the upcoming 7 days were created using physical activity data from the preceding 2 weeks. Machine learning models (elastic net [EN], random forest [RF], and neural network [NN]) were trained and validated on a 70:30 split cohort. Model performance was evaluated using the AUC.

**RESULTS:**

Among the 76 patients analyzed (median age 61 [IQR, 53-69] years, 39 [51%] female), 11 (14%) were hospitalized during the study period. The median step count during the first week of systemic treatment was 4,303 [IQR, 1926-7,056]. Unplanned hospitalizations in the upcoming 7 days could be predicted with high accuracy using RF (AUC = 0.88), NN (AUC = 0.84), and EN (AUC = 0.83). The models could not predict treatment modifications (AUC = 0.28-0.51) or the occurrence of any clinically relevant adverse event (AUC = 0.32-0.50).

**CONCLUSION:**

A decline in daily step counts can serve as an early predictor for hospitalizations in the upcoming 7 days, facilitating proactive and preventive toxicity management strategies.

## INTRODUCTION

In high-income countries, up to one in two people will develop cancer in their lifetime.^1^ The introduction of novel screening and treatment options has resulted in declined mortality rates.^2^ Cancer treatment can include surgery, radiotherapy, systemic treatment, or combinations of treatments. Often, patients require a multimodal approach using an individualized treatment strategy with the goal of balancing survival and health-related quality of life (HRQoL). These treatment modalities are frequently associated with toxicities. For example, 86% of patients treated with chemotherapy reported at least one toxicity during treatment,^3^ and 13% were hospitalized within 30 days of starting systemic treatment.^4^ Additionally, toxicities can lead to systemic treatment modifications, including dose reductions, treatment delays, or discontinuation,^5^ affecting treatment efficacy.^6,7^

**CONTEXT**

**Key Objective**
Are smartphone-measured step counts predictive for clinical outcomes during systemic cancer treatment?
**Knowledge Generated**
Machine learning models using step count data of the two preceding weeks were able to predict hospitalization in the upcoming week for patients during systemic therapy, but not other adverse events. Continuous monitoring could detect subtle changes in daily steps, which can serve as early indicators of health deterioration requiring hospitalization. Future studies should investigate whether this approach could enable proactive management strategies, preventing hospital admissions.
**Relevance *(P.P. Yu)***
Machine learning models incorporating decreases from baseline physical activity level, as measured by smartphone step counts, may predict for hospitalizations after chemotherapy.*
*Relevance section written by *JCO CCI* Associate Editor Peter Paul Yu, MD, FACP, FASCO.

Continuous symptom monitoring during treatment through electronic patient-reported outcomes (ePROs) has been demonstrated to enhance HRQoL,^8^ reduce symptom burden,^9^ and improve survival.^10^ Wearable devices allow for passive collection of real-time data vital signs, including step counts.^11^ Smartphones are also wearable devices equipped with multisensor systems, which are capable of continuously monitoring daily step counts. The use of smartphone-measured step count data would facilitate logistics and clinical implementation, compared with other objective devices such as accelerometers or pedometers, thereby reducing patient burden.^12^ Ambulatory monitoring of daily step counts of patients with cancer with smartphones has been demonstrated to be feasible in different treatment settings.^13-15^ Several studies among various cancer populations have shown that a decline in objectively measured step counts was associated with clinical outcomes, such as toxicities,^16,17^ hospitalizations,^18^ and progression-free survival.^14^ However, these studies were performed in specific cancer populations or used researcher-provided devices, hampering generalizability.

The upcoming availability of artificial intelligence and machine learning provides ample opportunities to analyze the wealth of objective and dynamic step count data to be leveraged in ways not previously possible.^19,20^ Identifying whether patterns in step counts can predict upcoming adverse events, such as hospitalization or treatment modifications, would be a crucial first step in using step counts for clinical monitoring. Recently, a machine learning–based model using step count measured with a wearable fitness tracker was found to predict hospitalization in patients receiving chemoradiation.^21^ However, it is unclear whether these results can be translated to a heterogeneous cancer population receiving systemic treatments using simple devices that patients already possess, such as smartphones. Continuous monitoring of daily step count could facilitate early preventive (supportive care) strategies, ultimately helping to optimize treatment and HRQoL for patients with cancer.

The aim of the STAPPS (Systemic Treatment related toxicity identified with a smartphone APPlication measuring Step count) study was to investigate whether changes in step counts measured with patients' own smartphone are predictive for an upcoming clinical adverse event (treatment modifications and hospitalizations), in a mixed population of patients receiving systemic anticancer treatments. In this paper, we explored the potential of using machine learning models to signal the occurrence of adverse events in the upcoming week.

## METHODS

### Study Population and Design

In this prospective observational cohort study (ClinicalTrials.gov identifier: NCT05927636), patients referred for systemic treatment at the departments of Medical Oncology at three hospitals (Radboudumc, Medisch Centrum Leeuwarden, and Erasmus MC) between September 2022 and February 2024 were recruited. Adult patients with a confirmed diagnosis of cancer were eligible if they were scheduled to start systemic treatment and possessed an Apple smartphone (the application appeared not yet compatible with Android smartphones during the study period). Exclusion criteria were cognitive disorders or severe emotional instability, physical impairments, limited walking ability (eg, paraplegia), and participation in an intervention study to promote physical activity. Ethical approval was waived by the Medical Ethics Committee of east Netherlands, and institutional review boards from participating hospitals approved the study. Before participation, all patients signed informed consent.

### Smartphone Measurements

Daily step counts were measured continuously throughout systemic treatment using patients' own smartphone with a developed application (OncoSTAPP, Brightfish, the Netherlands, available for free in the AppStore). After installing the application, patients received a pseudonymized code to activate the application. The application collects the daily step counts on the basis of the smartphones' built-in gyroscope, accelerometer, and global positioning system, and only sends the pseudonymized code, day, and daily step count to a secured database, thereby safeguarding privacy-sensitive data for application manufactures. Patients were given an instruction manual for installation, with contact details for any questions, and were instructed to carry their smartphone with them during all waking hours. Data collection was initiated at the start of systemic treatment and continued until completion or when the measuring period of 90 days was reached. A wear day was considered valid if any steps were recorded. At least two consecutive weeks of valid data were required for data analyses.

### Clinical Outcomes

Clinical adverse events were defined as any unplanned hospitalization or treatment modification and were retrieved from the patients' medical records. Dates of clinical adverse events were collected to calculate the day of the event during systemic treatment. Additionally, we collected the reason for hospitalizations or emergency department visit. Treatment modifications were defined as delays (≥1 week), dose reductions (≥25%), or (temporary) discontinuation of systemic treatment. Dates of treatment modification were set at the originally planned day for delays, the administration day for dose reductions, and the actual day of discontinuation.

### Covariables

Age, sex, Eastern Cooperative Oncology Group performance status, baseline comorbidity level, primary tumor type, and systemic treatment were retrieved from medical records. The comorbidity level was assessed using the Charlson comorbidity index^22^ and defined as elevated if the score was ≥1 after subtracting the primary tumor.

### Model Development

In line with previous methods,^21^ machine learning models were developed to predict clinical adverse events in the upcoming 7 days using step count data of the preceding 2 weeks. To reduce the impact of normal day-to-day variation and to handle missing data, daily step counts were smoothed using a 3-day running average,^21^ where each day's value was averaged with the previous 2 days. It has been described that at least 3 days of step count data are needed to obtain estimates of habitual step counts.^23^ The smoothed step counts from the two preceding weeks were then summarized into weekly features (mean, median, minimum, maximum, range, and standard deviation) and absolute and relative changes between features were calculated. The final database included all these aggregated features along with the smoothed step counts from the previous seven days. In case features were not computable because of missing data, this day was not included. If a patient was admitted to the hospital, the step counts during hospitalization was regarded as missing. Each day with sufficient data of the two preceding weeks was entered individually in the final database to develop models predicting an adverse event in the upcoming 7 days.

Patients were split into a training cohort and a testing cohort, with the first 70% of included patients allocated to the training cohort and the final 30% allocated to the testing cohort.^24^ To assess the robustness of the models, we performed a sensitivity analysis using 10 random 70:30 splits of the data. Model selection was based on promising results of a previous study among patients with cancer receiving chemoradiation and included an elastic net–regularized logistic regression (EN), a random forest (RF), and an ensembled sparse-input neural network (NN) model. These three machine learning models have different strengths and limitations. EN models combine traditional regression with regularization techniques to identify the most relevant features predicting the outcome while controlling for multicollinearity. However, this model is less effective at capturing complex, nonlinear relationships in the data.^25^ RF classification consists of multiple decision trees that collectively improve prediction accuracy and highlight important features. This model is robust and handles complex data sets well, but it can be computationally intensive and requires fine-tuning to prevent overfitting.^25^ NN models excel at detecting intricate patterns in large data sets by automatically extracting features from raw data. However, their decision making process is less transparent, making them harder to interpret in clinical settings where explainability is important.^25^ The area under the receiver operating characteristic AUC was used to evaluate the model on the testing cohort. Models were trained separately for the following outcomes: all clinical adverse events, hospitalizations, and treatment modifications.

Hyperparameters of machine learning models were tuned using grid search and 3-fold cross-validation (EN: L1 ratio and C; RF: maximum features and minimum samples split; and NN: hidden layer sizes, input, and full tree penalties). A Youden cutoff, optimizing both the sensitivity and specificity, was determined to assess specificity, sensitivity, and accuracy. An AUC above 0.8 was considered good for predicting the clinical outcomes.^26^ If the AUC was above 0.8, the feature coefficients of EN or the feature importance of RF was shown to aid in understanding the machine learning model.

All analyses were performed with R (version 4.2.2), RStudio software (version 2024.04.1, RStudio Team, PBC, Boston, MA), and Python (version 3.11.9, Python Software Foundation).

### Ethics Approval and Consent to Participate

All participants provided written informed consent before participating. Ethical approval was waived by the Medical Ethics Committee of east Netherlands, and institutional review boards from participating hospitals approved the study.

## RESULTS

**FIG 1.**
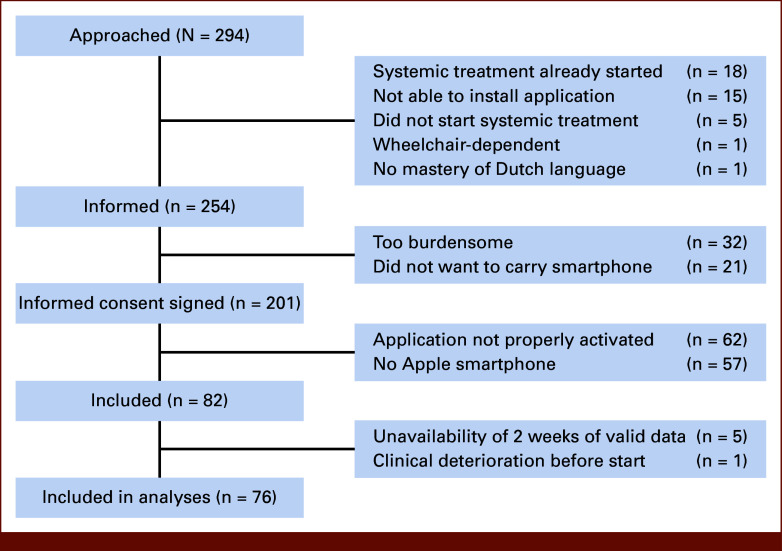
Flowchart of patients in the STAPPS study.

Of the 254 patients informed about the study, 201 (79%) signed informed consent (Fig 1). Main reasons for nonparticipation were that patients found the study too burdensome (13%) or did not want to carry a smartphone continuously (8%). For 31% of the patients who signed informed consent, the application was not properly activated and 28% did not possess an Apple device. Data from 76 patients (30% of informed patients) were included in the analyses.

The median age of the patients was 61 (IQR, 53-69) years, 39 patients (51%) were female, and 29 (38%) had metastatic disease (Table 1). The most common cancer type was breast cancer (27%) and chemotherapy was the most prescribed systemic treatment (55%). Eleven (14%) patients were hospitalized during the first 90 days of systemic treatment and 36 (47%) patients had at least one treatment modification. The main reason for hospitalization was an infection (n = 4/11; Table 2).

**TABLE 1.** Baseline Characteristics of Patients in the STAPPS Study

Characteristic	Total N = 76	Train n = 54	Test n = 22
Age, years, median (IQR)	61 (53-69)	65 (54-72)	55 (46-61)
Sex, No. (%) of women	39 (51)	26 (48)	13 (59)
Charlson comorbidity elevated (≥1, primary malignancy excluded), No. (%)	19 (25)	13 (24)	6 (27)
ECOG PS, No. (%)			
0	66 (87)	46 (85)	20 (91)
1	10 (13)	8 (15)	2 (9.1)
No previous cancer treatment, No. (%)	32 (42)	19 (35)	13 (59)
Metastatic cancer, No. (%)	29 (38)	22 (41)	7 (32)
Systemic treatment category, No. (%)			
Chemotherapy (±targeted)	42 (55)	27 (50)	15 (68)
Immunotherapy	6 (8)	4 (8)	2 (9)
Targeted therapy	6 (8)	6 (11)	0 (0)
Combination chemotherapy and immunotherapy	6 (8)	5 (10)	1 (5)
Systemic treatment combined with radiotherapy	16 (21)	12 (21)	4 (18)
Tumor types, No. (%)			
GI	22 (29)	18 (33)	4 (18)
Breast	21 (27)	15 (28)	6 (27)
Gynecologic	11 (14)	5 (9)	6 (27)
Head and neck	8 (11)	7 (13)	1 (5)
Genitourinary	8 (11)	5 (9)	3 (13)
Skin	4 (5)	3 (6)	1 (5)
Brain	2 (3)	1 (2)	1 (5)
Physical activity in the first week, median (IQR), step count per day	4,303 (1,926-7,056)	2,857 (1,395-6,227)	4,613 (2,170-7,438)
Hospitalization, No. (%) yes	11 (14)	8 (15)	3 (14)
Treatment modification, No. (%) yes	36 (47)	27 (50)	9 (41)

Abbreviation: ECOG PS, Eastern Cooperative Oncology Group performance status.

**TABLE 2.** Reasons for Hospitalizations

Reason	Total N = 11	Training n = 8	Test n = 3
Infection, No.	4	3	1
Dyspnea, No.	2	2	
Gastrointestinal toxicity, No.	2	2	
Fracture,^a^ No.	2	1	1
Thrombotic event, No.	1		1

a Hip fracture; pathologic fracture of humerus.

The median step count during the first week of systemic treatment was 4,303 (IQR, 1,926-7,056). Data from the 76 patients yielded a total of 4,544 possible prediction days, of which 1,061 prediction days (23%) were excluded because of missing data or hospitalization.

**FIG 2.**
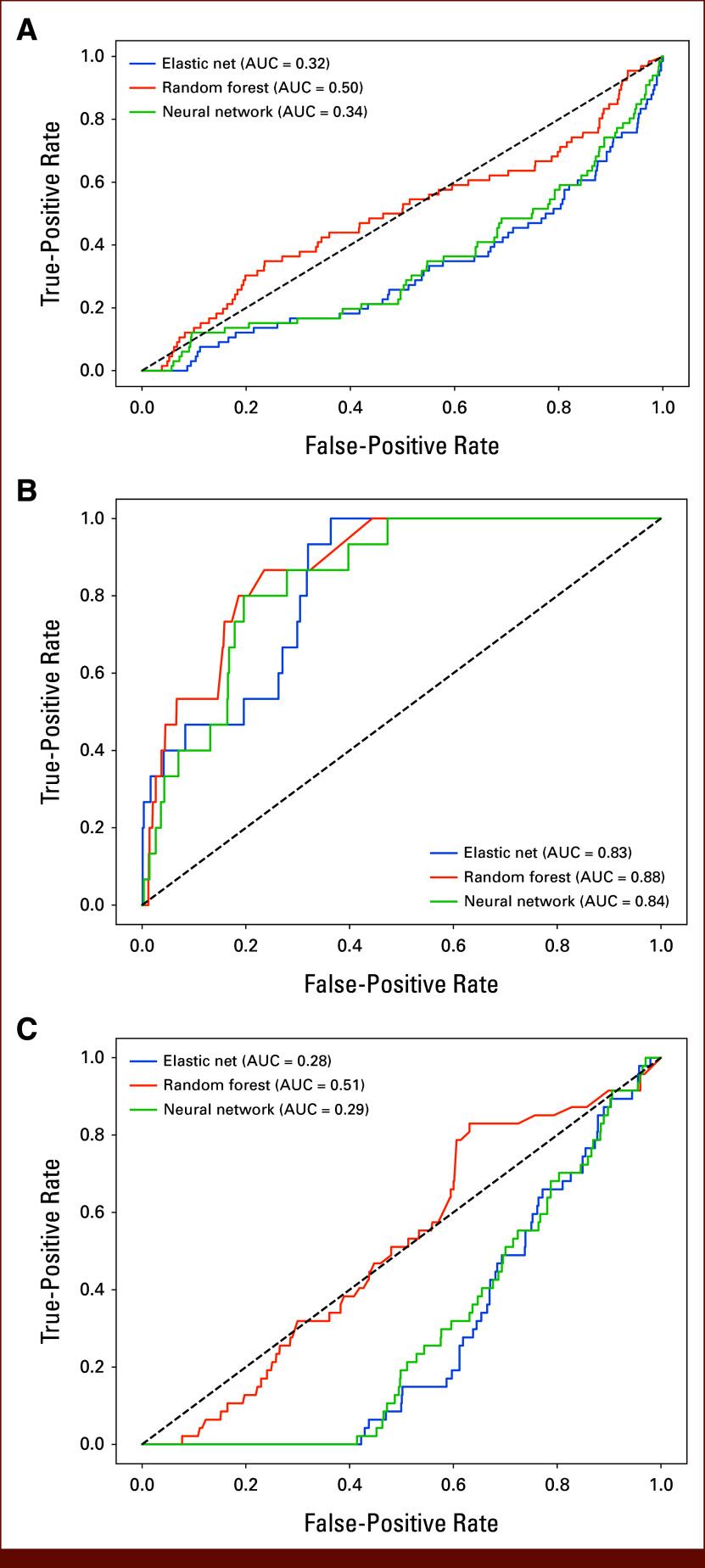
ROC curves of machine learning techniques, elastic net (blue), random forest (orange), and neural network (green), to predict (A) combined outcome, (B) hospitalizations and (C) tretment modifications. ROC, receiver operating characteristics.

None of the three machine learning techniques was able to predict all clinical adverse events together (AUC = 0.32-0.50; Fig 2A) nor treatment modification alone (AUC = 0.28-0.51; Fig 2C). All three models were able to predict hospitalizations in the upcoming 7 days using physical activity data from the preceding 2 weeks. RF showed the best predictive performance (AUC = 0.88; sensitivity = 87%; specificity = 76%; accuracy = 77%), followed by NN (AUC = 0.89; sensitivity = 80%; specificity = 80%; accuracy = 80%) and EN (AUC = 0.83; sensitivity = 100%; specificity = 64%; accuracy = 64%; Fig 2B). Sensitivity analyses revealed broad ranges in AUC (RF = 0.45-0.87; NN = 0.45-0.90; EN = 0.49-0.64).

**FIG 3.**
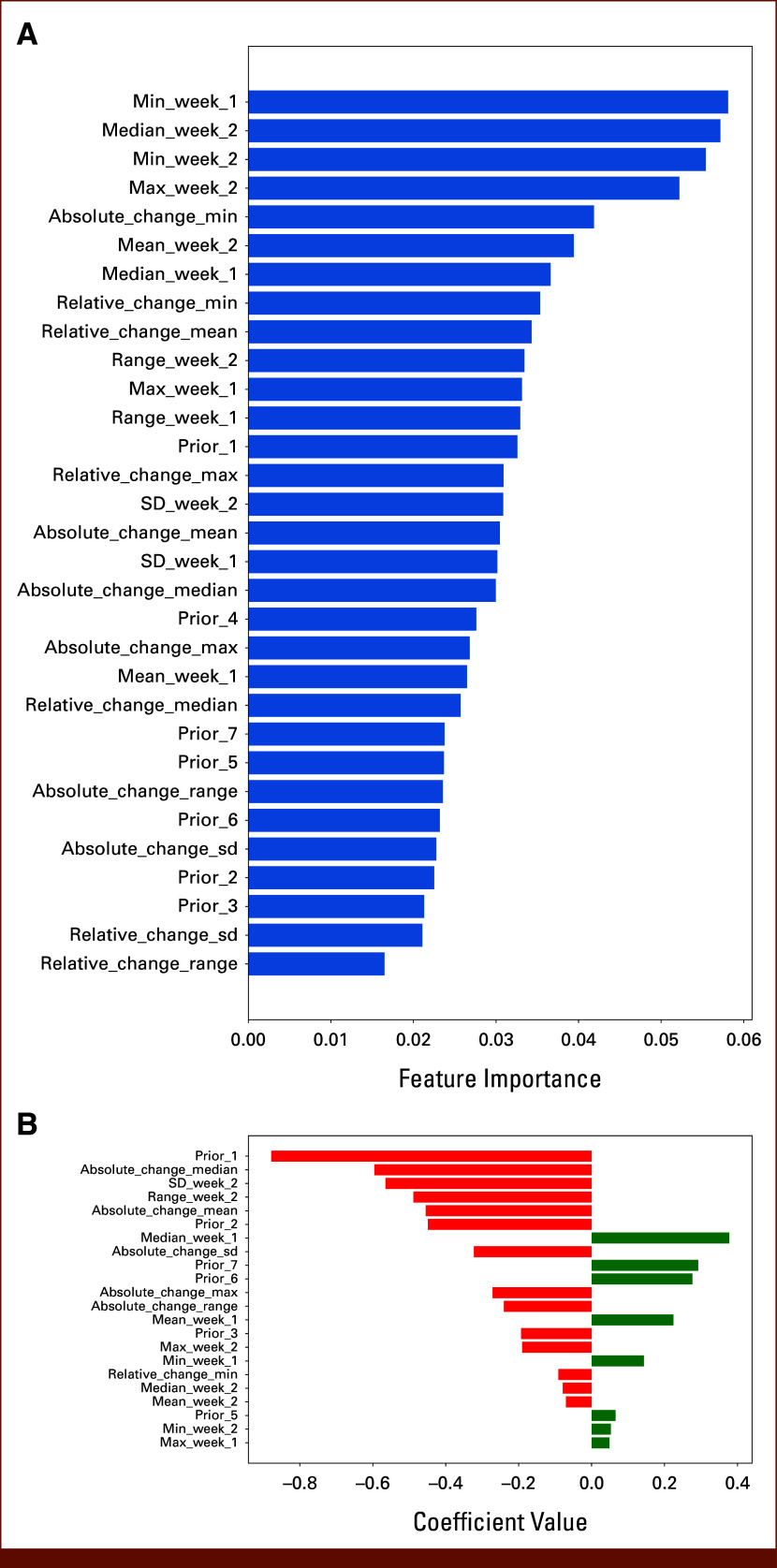
(A) Feature importance for random forest and (B) feature coefficients for elastic net for prediction hospitalizations.

In the RF model, the minimum step counts in the two preceding weeks and the median step count in the preceding week were identified as the most important features for predicting an upcoming hospitalization within 7 days (Fig 3A). The EN model identified the smoothed step counts before the prediction day and the absolute change in median number of steps between weeks as the most influential features (Fig 3B).

## DISCUSSION

This study explored the potential of machine learning models to predict clinical adverse events using daily step counts measured with patients' own smartphones. The machine learning models used in this study were able to predict hospitalizations in the upcoming 7 days using daily step counts from the preceding 2 weeks, with a good predictive performance. The RF model demonstrated the highest predictive performance. The models were not able to predict the occurrence of any clinical adverse event and upcoming treatment modifications.

Our finding that patterns in daily step count can signal the likelihood of hospitalization within the following 7 days underscores the potential of continuous monitoring during systemic cancer treatment. These results align with a previous study, demonstrating high predictive value of step counts for hospitalizations in patients undergoing chemoradiation.^21^ Accurate and timely prediction of hospitalizations could open avenues for early interventions, to prevent unplanned hospitalizations in patients receiving systemic cancer treatment.^27^ The specific causes for hospitalization varied and could be due to a combination of therapy-related effects, disease progression, or exacerbation of preexisting conditions, which is difficult to disentangle. Reductions in step count may serve as a general indicator of increased risk of hospitalization. Future studies with larger sample size should reveal whether predictions vary across different causes of hospitalization. Yet, findings showed that daily step counts could serve as a passive measurement to alert upcoming adverse events. Like ePROs used for symptom monitoring that have been shown to reduce adverse events, including hospitalizations^28^ and to improve HRQoL,^8,29^ integration of daily step count monitoring into clinical practice could prompt timely patient contact and targeted interventions to address underlying issues and prevent hospitalizations.

In contrast to hospitalizations, the occurrence of any adverse events and treatment modifications alone could not be predicted using step count data. A potential explanation for this could be that not all decisions that result in a treatment modification are based on factors affecting daily activity. This includes asymptomatic reasons such as abnormal laboratory values (eg, neutropenia), resulting in dose delays.^30^ Second, different types of cancer and systemic treatments each have distinct toxicity profiles, which can affect patients' daily activity in various ways. This heterogeneity leads to wide variability in daily step counts before toxicities, making prediction modeling more challenging. In comparison, physical activity patterns preceding hospitalizations may be more consistent across cancer types and treatments, enabling more reliable predictions using this digital biomarker. Furthermore, the onset of treatment toxicity may have occurred earlier than the actual date of treatment modification as initiated by the oncologist during the regular visits to the outpatient clinic for the systemic treatment. Using ePROs to monitor adverse events could enhance timely and accurately detection of treatment-related toxicity,^31,32^ facilitating better exploration of the predictive value of step counts.

Different machine learning models were evaluated for predicting adverse events, all demonstrating good predictive performance. This confirms that the relationship between daily step counts and hospitalization can be identified using multiple models. Yet, the optimal method and the robustness of the models need to be established. The present models were based on various combinations of step count data in the days or weeks before the event, and on computed changes in step counts over preceding 2 weeks. This highlights that no single value or threshold can predict hospitalizations in the coming week, which was previously suggested.^16,17^

Strengths of the current study include the utilization of patients' own smartphones, facilitating easy, objective, and unobtrusive measurements of physical activity in a patients' home environment at a large scale.^33^ Since nearly all smartphones are already equipped to collect these data, there is no need for specialized health-grade devices, avoiding additional costs and reducing the burden on clinicians. Furthermore, the prospective study design, the focus on a heterogeneous cancer population, and a relatively long measuring period of 90 days on systemic treatment enhance the generalizability of the findings.

Several limitations should be considered when interpreting these results. First, the relatively low number of patients and events can increase the risk of overfitting in the models, potentially limiting their robustness. This was also reflected by the broad range of AUCs when random allocation to 70/30 splits were used. Additionally, the low event rate limits evaluating the models' clinical utility. Although the results highlight the potential of machine learning, further validation in larger populations is essential before clinical implementation. Second, data on nonwear time of the smartphone are currently lacking. Although patients were instructed to always carry the phone during waking hours, we could not differentiate between low step count and nonwear time.^34^ The increasing availability and use of smartwatches, enabling simultaneous heart rate monitoring, could enhance the modeling and help account for nonwear time. However, nonwear time itself could also indicate clinical deterioration, as patients who feel unwell often use their phones less.^13,35^ Additionally, using step count as the sole measure of physical activity may underestimate total activity levels, as it does not capture activities such as cycling or swimming.^36^ Yet, step count itself may be a good indicator of general health as it is associated with many clinical outcomes.^36,37^ Third, although the study initially aimed to include an Android version of the application, technical issues prevented its use, potentially introducing selection bias toward patients from higher socioeconomic backgrounds.^38^ Additionally, in 31% of patients, the application was not properly activated, which could be due to technical problems or patients not activating the application and thus declining study participation. Ensuring the availability of a device or application that is intuitive and accessible to all patients is critical for its acceptance and effective utilization in clinical practice.

The integration of machine learning into clinical decision making is becoming increasingly popular,^39^ and regulatory agencies are beginning to recognize digital biomarkers as primary end points for assessing treatment efficacy.^40^ This progress creates a favorable environment for incorporating digital monitoring and machine learning–based prediction models into clinical workflows. Future research should further explore the potential of wearable device monitoring in predicting and preventing adverse events. Our data suggest that step counts alone may not capture all relevant information for predictions of all clinical outcomes. Integrating additional wearable device metrics such as physical activity intensity, activity type, circadian rhythms, sleep duration, heart rate, and heart rate variability^33^ could provide a more comprehensive assessment of a patient's health. Future studies should reveal whether incorporating these metrics alongside clinical data would improve predictions of clinical outcomes. Beyond model development, future studies should evaluate the impact of using such models in preventing adverse events. Similar to evaluations of patient-reported symptom monitoring,^8,29^ (cluster) randomized controlled trials or stepped-wedge trials should determine the clinical value.

In conclusion, machine learning models using step counts measured with a smartphone during systemic anticancer treatment demonstrated predictive value for the occurrence of hospitalizations in the upcoming week, but not for other adverse events. These machine learning approaches can leverage the continuous monitoring capability of smartphones to detect subtle changes in physical activity, which can serve as early indicators of health deterioration requiring hospitalization. Future studies should investigate whether integrating predictive models into clinical practice can enable proactive management strategies, ultimately improving patient HRQoL and treatment outcomes.

## Data Availability

The data sets used and/or analyzed during the current study are available from the corresponding author on reasonable request.
